# Supplementary material for: The Synergistic Effect of MoS2 and NiS on the Electrical Properties of Iron Anodes for Ni-Fe Batteries
Source: Nanomaterials (Basel). 2022 Oct 4;12(19):3472. doi: 10.3390/nano12193472 (PMC9565254; doi:10.3390/nano12193472)
Supplement: Supplementary file 1 [file nanomaterials-12-03472-s001.zip › nanomaterials-1934608-supplementary.pdf]

nanomaterials

MDPI

Supplementary Materials

The Synergistic Effect of MoS_2 and NiS on the Electrical Properties of Iron Anodes for Ni-Fe Batteries

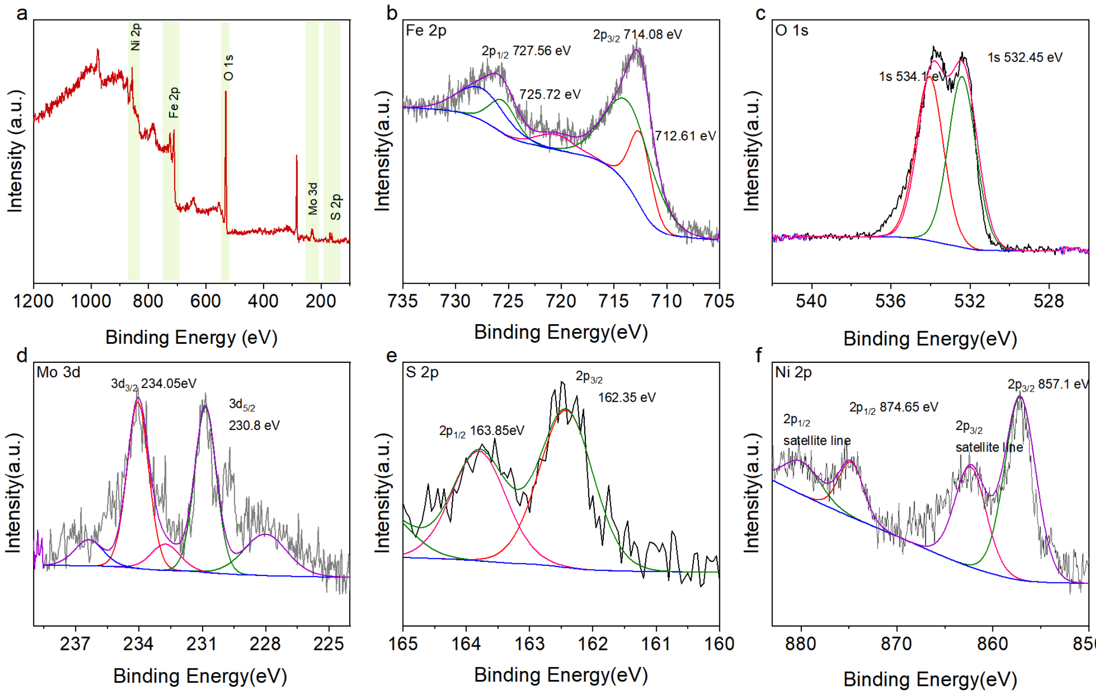

Figure S1. XPS results of Fe_3O_4/MoS_2/NiS (5%). (**a**) Wide-range scanning result of XPS spectrum. High-resolution XPS spectrum of Fe 2p (**b**), O 1s (**c**), Mo 3d (d), S 2p (**e**) and Ni 2p (**f**).

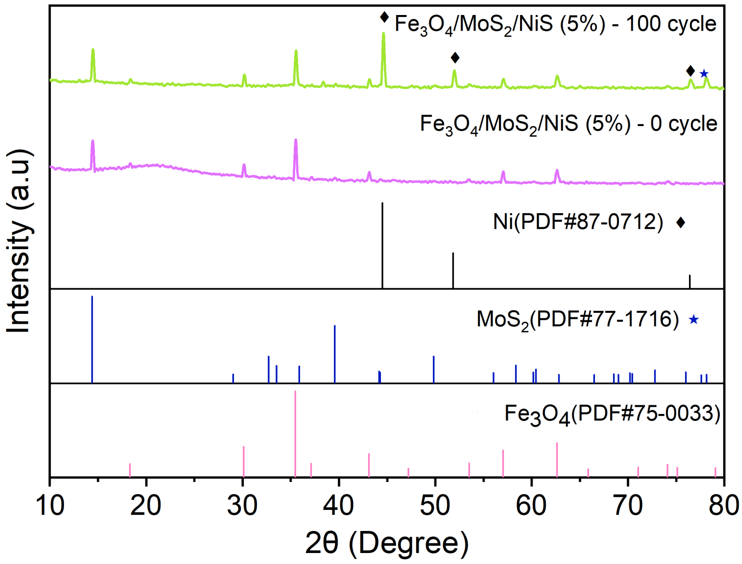

Figure S2. XRD patterns of the Fe_3O_4/MoS_2/NiS (5%) sample before cycle and after 100 cycles.

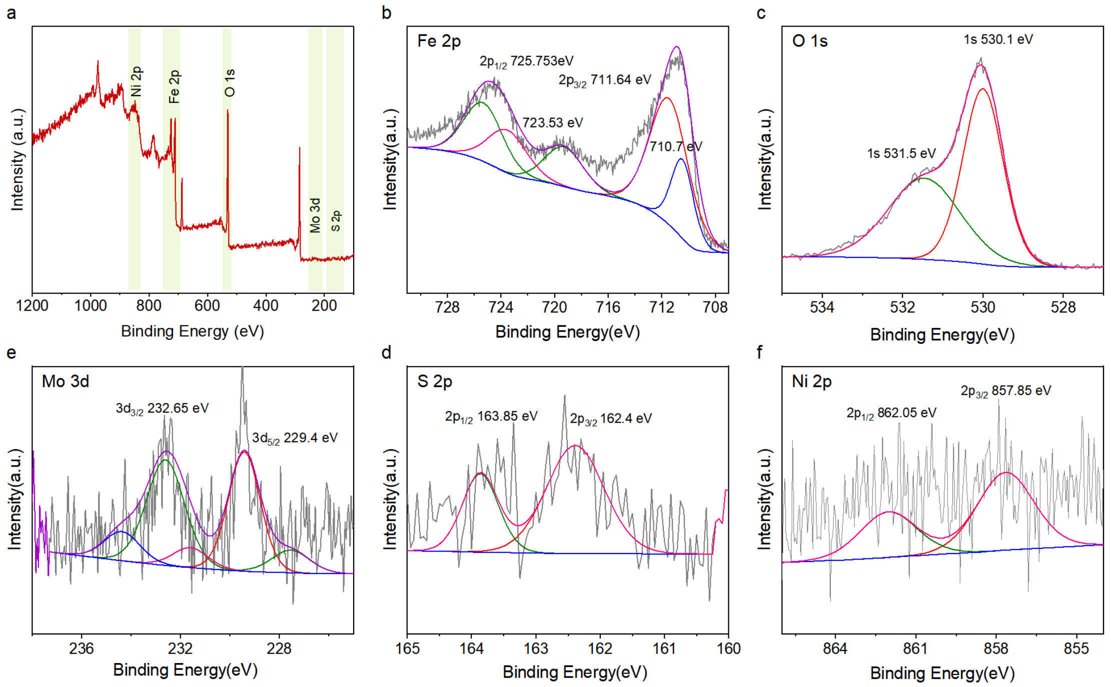

Figure S3. XPS results of $Fe_3O_4/MoS_2/NiS$ (5%) samples after 100 cycles. (**a**) Wide-range scanning result of XPS spectrum. High-resolution XPS spectrum of Fe 2p (**b**), O 1s (**c**), Mo 3d (**d**), S 2p (**e**) and Ni 2p (**f**).

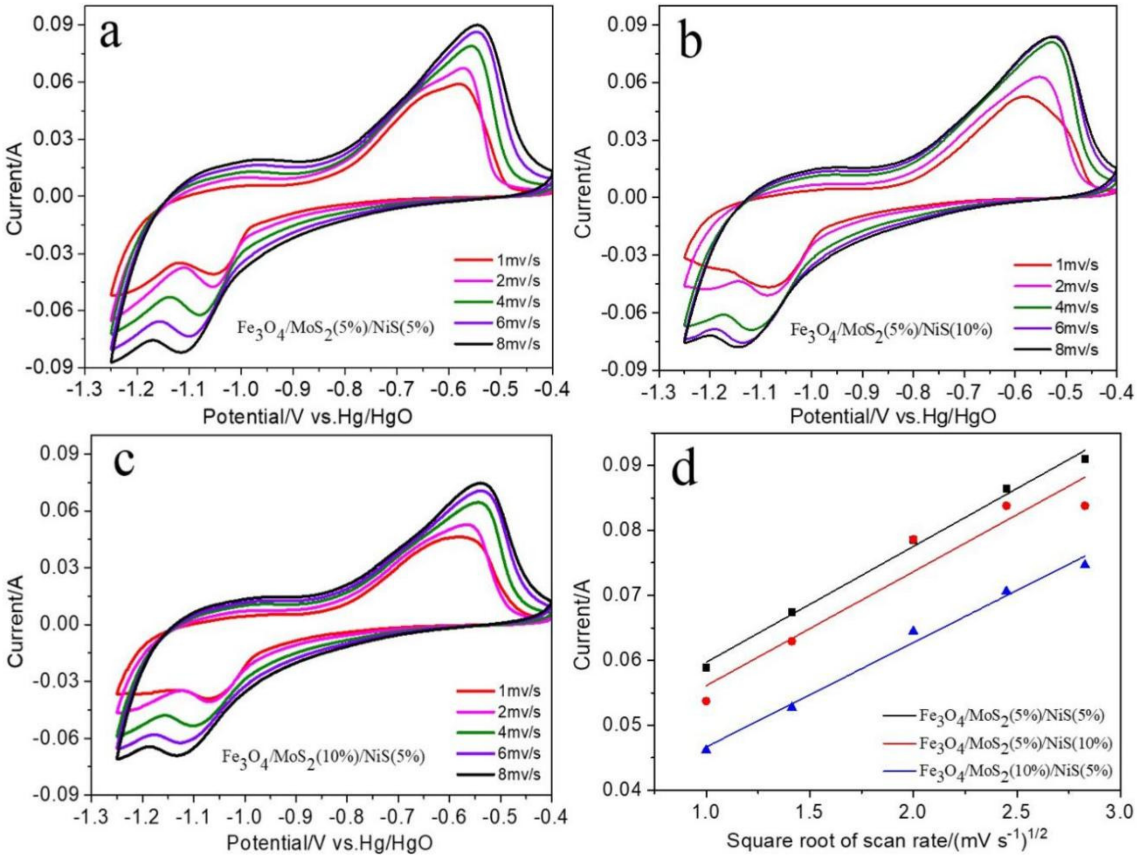

Figure S4. (**a**-**c**) Cyclic voltammetry curves of three samples; (**d**) relationships between the maximum oxidation peak currents of the three electrodes and the square root of the scanning rate.

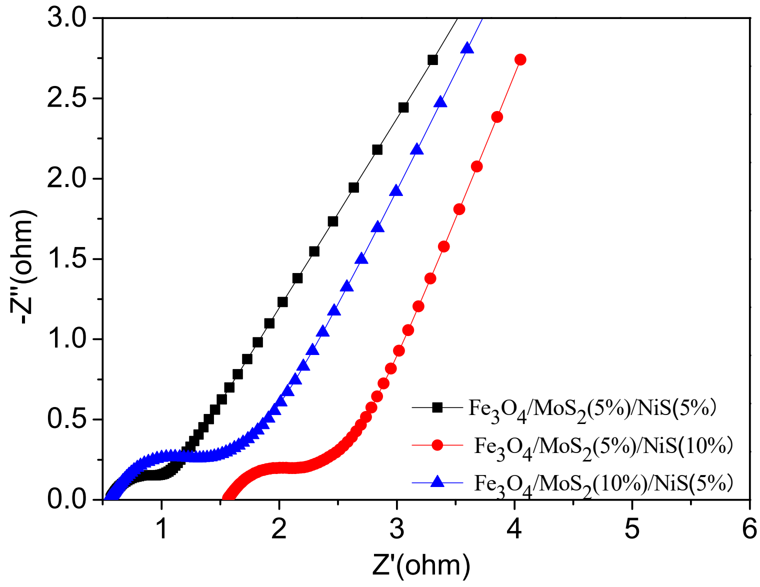

Figure S5. AC impedance spectra of the Fe_3O_4/MoS_2 (5%)/NiS (5%), Fe_3O_4/MoS_2 (5%)/NiS (10%) and Fe_3O_4/MoS_2 (10%)/NiS (5%) electrodes.

Table S1. Anode and cathode potentials and potential differences of three sample electrodes.

Electrode	E_O (mV)	E_R (mV)	$\triangle E_{O\text{-}R}$ (mV)
Fe_3O_4/MoS_2 (5%)/NiS (5%)	583	1053	470
Fe_3O_4/MoS_2 (5%)/NiS (10%)	582	1080	498
Fe_3O_4/MoS_2 (10%)/NiS (5%)	576	1075	499
